# A Short History of Clinical Holistic Medicine

**DOI:** 10.1100/tsw.2007.238

**Published:** 2007-10-05

**Authors:** Søren Ventegodt, Isack Kandel, Joav Merrick

**Affiliations:** ^1^ Quality of Life Research Center, Teglgårdstræde 4-8, DK-1452 Copenhagen K, Denmark; ^2^ Research Clinic for Holistic Medicine, Copenhagen, Denmark; ^3^ Nordic School of Holistic Medicine, Copenhagen, Denmark; ^4^ Scandinavian Foundation for Holistic Medicine, Sandvika, Norway; ^5^ Interuniversity College, Graz, Austria; ^6^ Faculty of Social Sciences, Department of Behavioral Sciences, Ariel University Center, Samaria, Ariel, Israel; ^7^ National Institute of Child Health and Human Development, Jerusalem, Israel; ^8^ Office of the Medical Director, Division for Mental Retardation, Ministry of Social Affairs, Jerusalem, Israel; ^9^ Kentucky Children's Hospital, University of Kentucky, Lexington, USA

**Keywords:** Holistic health and medicine, complementary and alternative medicine

## Abstract

Clinical holistic medicine has its roots in the medicine and tradition of Hippocrates. Modern epidemiological research in quality of life, the emerging science of complementary and alternative medicine, the tradition of psychodynamic therapy, and the tradition of bodywork are merging into a new scientific way of treating patients. This approach seems able to help every second patient with physical, mental, existential or sexual health problem in 20 sessions over one year. The paper discusses the development of holistic medicine into scientific holistic medicine with discussion of future research efforts.

## INTRODUCTION

Millennia ago, around 300 BCE, at the island of Cos in old Greece, the students of the famous physician Hippocrates (460-377BCE)[1] worked to help their patients to step into character, get direction in life, and use their human talents for the benefit of their surrounding world. For all that we know this approach was extremely efficient medicine that helped the patients to recover health, quality of life, and ability, and Hippocrates gained great fame. For more than 2,000 years this was what medicine was about in most of Europe.

On other continents similar medical systems were developed. The medicine wheel of the native Americans, the African Sangoma culture, the Samic Shamans of northern Europe, the healers of the Australian Aboriginals, the ayurvedic doctors of India, the acupuncturists of China, and the herbal doctors of Tibet all seems to be fundamentally character medicine[2,3,4,5,6,7,8]. All the theories and the medical understanding from these pre-modern cultures are now being integrated in what is called integrative or transcultural medicine. Many of the old medical systems are reappearing in modern time as alternative, complementary and psychosocial medicine. This huge body of theory is now being offered as a European Union Master of Science degree[2,3,4,5,6,7,8].

## WHAT IS HAPPENING TODAY?

Interestingly, two huge movements of the last century have put this old knowledge into use: psychoanalysis[9] and psychodynamic therapy[10,11] (most importantly STPP[12,13]) going though the mind on one hand, and bodywork (most importantly Reich[14], Lowen[15] and Rosen[16]) and sexual therapy (especially the tantric tradition[17]) going through the body on the other. A third road, but much less common path has been directly though the spiritual reconnection with the world[18,19].

Our international research collaboration got interested in existential healing from the data coming from epidemiological research at the University Hospital of Copenhagen (Rigshospitalet) starting in 1958-61 at the Research Unit for Prospective Paediatrics and the Copenhagen Perinatal Birth Cohort 1959-61. Almost 20 years ago we were conducting epidemiological research on quality of life, closely examining the connection between global quality of life and health for more than 11.000 people in a series of huge surveys[see 20] using large and extensive questionnaires, some of them with over 3,000 questions. We found (quite surprisingly) from this huge data base that quality of life, mental and physical health, and ability of social, sexual and working ability seemed to be caused primarily by the consciousness and philosophy of life of the person in question, and only to a small extent by objective factors, like being adopted, coming from a family with only one breadwinner, mother being mentally ill, or one self being financially poor or poorly educated (which are obviously very much socially inherited)[20].

This scientific finding was not expected and so contra-intuitive for us that we were forced to investigate the subject going to the roots of western medicine, or the Hippocratic character of medicine. This meant that we had to look at transcultural and integrative medicine, the emerging science of alternative medicine (scientific CAM theory) and to the very much forgotten traditions of psychosomatic, psychodynamic, and bodily oriented therapies. Around 1994 we received substantial fundings for our research project trying to embrace this huge heritage of medical wisdom philosophically[21,22,23,24,25,26,27,28], theoretically [29-49], epidemiologically/statistically [50-71].

We have since 1997 with a great effort tried to take this knowledge into clinical practice [72-113], and with quite extraordinary results. Clinical holistic medicine has in our Research Clinic for Holistic medicine in Copenhagen helped every second patient with physical, mental, existential or sexual health issues or diseases over one year[114-119]. Finally we have been looking at what seems to be the common denominator for all existential healing work in all cultures at all times: the sense of coherence, most clearly expressed by Aaron Antonovsky (1923-1994), a sociologist from the Faculty of Health Sciences at the Ben Gurion University of the Negev in Israel[18,19,120-125]. We have also been debating many difficult issues related to modern day medical science, especially in the British Medical Journal [126-139] and finally we are now collecting most of what we consider essential knowledge for the holistic physician in a series of books on the “Principles of holistic medicine”[140-142].

What we have learned from this long journey through the grand medical heritage from the different cultures on this planet is that we need to work on body, mind and spirit at the same time (medicine men has always combined talking, touching, and praying), and that being human and truly kind is what really heals the other person. This is what Hippocrates called “the Art”[1], not “the art of medicine” or “the art of right living”, but simply “the art” – the way of the human heart, cultivating existence into sheer compassionate behaviour and joyful being, which has always been the ultimate goal of all the great healers in our history.

We are more than happy to see our research project in scientific holistic medicine (clinical holistic medicine, CHM) developing. The most paradoxal aspect of this is that while we like to think we are taking medicine forward, we are actually just taking medicine back to its roots.

The most important thing is that research and development in this field is made in a dialectic process between qualitative and quantitative research.

## QUALITATIVE AND QUANTITATIVE RESEARCH[139]

There are basically two ways of documenting an effect of a holistic medical intervention, the quantitative and the qualitative approach. Much effort has been given to developing valid methodology and measuring tools, but the art of documentation has become a complex and expensive task. Due to lack of resources we have been forced to seek simple, but still valid ways of documenting effect[75]. In this communication we will focus on the qualitative research method.

Fortunately the holistic approach makes it much simpler, because there are always three domains to investigate: health, quality of life (QOL) and ability. These three domains can be subdivided in as many detailed domains as one wishes, but often three are sufficient for most purposes.

There are two qualitative aspects of documenting effect in medicine, often called subjective (that is from the perspective and experience of the patient) - and objective (that is from the perspective of the therapist or researcher). To document effect of an intervention using both perspectives, the patient must be interviewed before and after the intervention. Semi structured interviews with interviewer rating of the state immediately before and after the intervention can be used to give the objective perspective on the effect of the intervention. Interviewing the patient after the intervention can give the patient's subjective experience of the effect.

Most importantly these perspectives often leads to two different results, but confronting the patient with the observed improvement, after the patient has given his own experience of the effect, can be very enlightening.

The consensus paradigm states that only to the degree that there is consensus between patient and therapist/observer, the treatment has an effect. If the patient experience an effect that cannot be observed, something else is likely to have happened, i.e. an upgrade of other dimensions than the three defined as outcome. Instead of QOL, health and ability the patient has gained self-esteem, confidence, admiration from others etc. As holistic medicine aims to improve life in these three domains, a pleasant experience with the therapy is not the same as en effect of a treatment.

If the patient does not experience an observed effect, this effect is most likely to be happening only in the observer’s mind. Very often a therapist is convinced that a cure or intervention gave a positive result, but the fact that the patient did not experience that is then often neglected. In holistic medicine the dimensions we want to improve are highly experiential, so if the patient did not experience any improvement, such an improvement is most likely not to have happened.

Interestingly one single patient is enough to document effect with the consensus paradigm. If both the physician and his patient, after careful investigation before and after the treatment, find that the treatment has helped, this is most likely the case. The more precise the target group and the treatment are defined the more valuable the documentation. We recommend for securing the validity that the presented method is used with five highly comparable patients receiving five highly comparable treatments.

As always we recommend for the observer rating a five point symmetrical Likert scale with neutral middle point and equidistance[143]. A clinically significant improvement must be half a step on this scale or more. The patient needs to express the gain as a "significant improvement". When both patient and observer find improvement of QOL, health, and ability significant (according to the above), we call the treatment "good".

## PERSPECTIVES FOR FUTURE RESEARCH AND DEVELOPMENT[28]

There are lots of possible advantages with the scientific holistic medicine that must be closely examined in future research:

- How can it be make a affordable, efficient medicine for the future
- The possibility to prevent disease

- The possibility to cure cancer and coronary heart disease
- The possibility to seroconvert HIV-positive patients to HIV negative
- The possibility to relief pain and discomfort
- The possibility of rehabilitating working ability
- The possibility of improving peoples competency as parents
- The possibility of improving working efficiency though development of talent
- The possibility of helping people to be happy in spite of difficult circumstances and challenges
- The possibility of people developing consciousness and becoming more responsible for local and global environment

## ACKNOWLEDGMENTS

The Danish Quality of Life Survey and the Quality of Life Research Center was 1991-2004 supported by grants from the 1991 Pharmacy Foundation, the Goodwill-fonden, the JL-Foundation, E. Danielsen and Wife's Foundation, Emmerick Meyer's Trust, the Frimodt-Heineken Foundation, the Hede Nielsen Family Foundation, Petrus Andersens Fond, Wholesaler C.P. Frederiksens Study Trust, Else & Mogens Wedell-Wedellsborg's Foundation and IMK Almene Fond. The research was approved by the Copenhagen Scientific Ethical Committee under number (KF)V.100.2123/91 and further correspondence.
